# Disease burden for patients with primary immunodeficiency diseases identified at reference hospitals in Guanajuato, Mexico

**DOI:** 10.1371/journal.pone.0175867

**Published:** 2017-04-27

**Authors:** Eduardo Guaní-Guerra, Ana Isabel Jiménez-Romero, Ulises Noel García-Ramírez, José Manuel Velázquez-Ávalos, Edgar Martínez-Guzmán, Eunice Sandoval-Ramírez, Ignacio Camacho-Meza

**Affiliations:** 1 Department of Immunology, Hospital Regional de Alta Especialidad del Bajío, León Guanajuato, México; 2 Department of Immuno-Alergology, Hospital Aranda de la Parra, León Guanajuato, México; 3 Departament of Immuno-Allergology Pediatrics, Hospital Ángeles-León, León Guanajuato, México; 4 Department of Immuno-Allergology, Centro Médico Nacional del Bajío, IMSS, León Guanajuato, México; 5 Department of Immuno-Allergology Pediatrics, Hospital Pediátrico de León, León Guanajuato, México; Universidade Nova de Lisboa Instituto de Higiene e Medicina Tropical, PORTUGAL

## Abstract

**Background:**

In addition to the deleterious effect on health, there is considerable economic and psychosocial morbidity associated with primary immunodeficiency diseases (PID). Also, the cost of a late diagnosis frequently results in a heavy disease burden on the patient. The objective of this study was to collect and analyze data on patients with PID in the state of Guanajuato in Mexico, to indirectly estimate the burden of the disease.

**Methods:**

An observational, longitudinal, and comparative study was conducted. A total of 44 patients were included and grouped according to the updated classification of PID.

**Results:**

The median time elapsed from the onset of symptoms to the reference and diagnosis by a tertiary hospital was of 2.17 (IQR = 6.44) years. Before diagnosis, the number of hospitalizations/year per patient was 0.86 (IQR = 2.28), the number of visit to emergency room/year per patient was 0.92 (IQR = 1.77), the number of doctor’s visits/year per patient was 15 (IQR = 11.25), whereas the school/work absence days per patient were reported in 52.72 (IQR = 56.35) days per year. After diagnosis, 20 patients (45.45%) received IVIG replacement therapy, and all of them presented a significant improvement (p <0.05) in all the mentioned variables. Characteristically, even when patients with PID received IVIG, there was still an important disease burden when comparing them against healthy controls. Complications secondary to PID were detected in 19 patients (43.18%). The reported overall mortality rate was 6.82% (n = 3).

**Conclusions:**

We were able to indirectly estimate an important disease burden in patients with PID; which is considered to be preventable, at least in part, with effective interventions like health planning, research, collaboration with primary care providers, and generation of policies and practices, in order to improve the quality of life and care of families with PID.

## Introduction

PIDs are a group of heterogeneous disorders with immune system abnormalities characterized by various combinations of recurrent infections, autoimmunity, lymphoproliferation, granulomatous process, atopy, and malignancy [1]. Over the last 65 years, the field of PIDs has advanced greatly. With the advent of cutting-edge genetic technology, more than 240 PIDs have been discovered and the number continues to increase [2].

These disorders are proven to be of higher incidence and prevalence than previously thought. PIDs are now appreciated to range from 1:500 to 1:500,000 in the general population in the United States and Europe [3,4]. A random digit dialing telephone survey in 2007 estimated that one in 1200 people within the United States are diagnosed with an immunodeficiency [5].

In addition to the deleterious effect on health, there is considerable economic and psychosocial morbidity associated with these disorders. Tragically, the cost of late diagnosis results in a heavy burden of disease on the patient [6,7]. Calculation of disease burden is necessary for research, resource allocation, and generation of policies and practices. Moreover, baseline burden facilitates the analysis of the cost-effectiveness of new interventions and programs [8].

In Mexico, we still lack of studies that show the burden and cost of PIDs in our population. The objective of the present study is to collect and analyze data on patients with PID in the state of Guanajuato in Mexico, to indirectly estimate the burden of the disease.

## Materials and methods

We conducted an observational, longitudinal and comparative study, in five different reference centers or tertiary referral hospitals in the state of Guanajuato, Mexico. Epidemiological and clinical data were obtained by review of clinical files. After this, authors interviewed patients or their relatives, and established a prospective data base using a standard questionnaire to obtain the following variables: family expenses, work/lost school days, mean time at the onset of symptoms, number of visits to emergency room, number of doctors’ visits, insurance coverage, health sector and parental consanguinity; data was obtained using a standard questionnaire. We also collected data regarding cost for day of hospitalization, visit to emergency room (ER), and visits to the physician’s office, directly from the accounting department and financial support of each participant hospital. A total of 44 patients were included and grouped according to the 2015 updated classification of PIDs introduced by the Expert Committee of International Union of Immunological Societies (IUIS) on Primary Immunodeficiency [2]. Diagnosis was made following the main and current international criteria [9][10]. The study subjects included in the present survey were patients from the Immunology Department of the different reference centers. The five referral hospitals, at the moment of the study, have a mean of 83,171 active patients; 147.25 hospital beds; 7,113.33 admissions per year; and 7,032.67 outpatients per year. Their influence area comprises the central-bajio region in Mexico, and gives service at least to 6 million people, mainly in the state of Guanajuato.

The patients’ parents were considered to be related if parental consanguinity was of the first or second degree. The study was approved by the ethics and research committee of the Hospital Regional de Alta Especialidad del Bajío. Participants included in the study provided their written informed consent. Quantitative variables were described using median with interquartile range (IQR), and mean ± standard deviation (SD). Categorical data were described using absolute and relative frequencies. Regarding to the comparative analysis between patients with IDP before and after treatment with intravenous immunoglobulin (IVIG), data were compared using Wilcoxon signed-rank test. We also compered patient with IDP before and after the treatment with IVIG, with healthy matched controls, using Mann-Whitney U test. The healthy matched controls were siblings or friends of the same gender and similar age, without PID or chronic diseases, except for one who had mild intermittent asthma. Statistical analysis was performed using the Sigmaplot v.12.0 software for Windows and VassarStats.

## Results

A total number of 44 patients were included in the study, 18 females (40.91%) and 26 males (59.09%). Male-to-female ratio was 1.44:1. The mean age at the beginning of the study was 11.64 ± 11.59 years (n = 41). The median time in diagnostic delay, defined as the time elapsed between the onset of symptoms and the moment of diagnosis, was of 2.17 years (IQR = 6.44) (Table 1).

**Table 1:** General characteristics of patients with primary immunodeficiency diseases.

	STUDY GROUP n = 44
Gender (Male/Female)	26/18
Age at the onset of symptoms in years, median (IQR)	1.12 (4.06)
Age at the time of diagnosis in years, median (IQR)	4.87(9.48)
Time in diagnostic delay in years, median (IQR)	2.17(6.44)
Patients under 16 years at the moment of diagnosis, n(%)	39 (88.64%)
Health sector, n(%)	
Public	34(77.27%)
Private	10(22.73%)
Parental consanguinity, n(%)	5(11.4%)
Number of patients with other comorbidities, n(%)	24(54.55%)
Allergic rhinitis	18(40.91%)
Asthma	8(18.18%)
Food allergy	4(9.09%)
Atopic dermatitis	3(6.82%)
Autoimmunity	4(9.09%)
Hypothyroidism	3(6.82%)
Cardiopathy	2(4.54%)
Epilepsy	2(4.54%)
Gastroesophageal reflux	2(4.54%)
Down syndrome	2(4.54%)
Others	3(6.82%)

IQR = interquartile range.

Regarding the health sector, where patients received medical care, 34 (77.27%) attended to public hospitals, and 10 study subjects (22.73%) belonged to private practice institutions. A history of known parental consanguinity was recorded in five cases (11.4%). No family history of primary immunodeficiency disease was reported, except for two siblings included in the present study, both with common variable immunodeficiency (CVID). A total of 24 patients with PID (54.55%) suffered from other serious, chronic disease. Among the comorbidities, the most frequent was allergic rhinitis (40.91%), followed by asthma (18.18%), food allergy (9.09%), autoimmunity (9.09%), atopic dermatitis (6.82%), hypothyroidism (6.82%), cardiopathy (4.54%), epilepsy (4.54%), gastroesophageal reflux (4.54%), and Down Syndrome (4.54%). Whereas, other diseases like lymphoma, pulmonary hypertension, and urticaria, were reported each one, in the 2.27% of the study subjects (Table 1).

Regarding to the spectrum of PID in our study population, the most prevalent disorder were the predominantly antibody deficiency diseases (63.64%); followed by combined immunodeficiencies with associated or syndromic features (13.64%), congenital defects of phagocyte number and/or function (9.1%), complement deficiencies (4.54%), defects in innate immunity (4.54%), combined immunodeficiencies (2.27%), and diseases of immune dysregulation (2.27%). Among the humoral immunodeficiencies, the most frequent were the common variable immunodeficiency disorders (11/28, 39.29%) (Table 2).

**Table 2:** Spectrum of PID at reference and high specialty hospitals in Guanajuato Sate.

PID	STUDY GROUP n = 44	SUPPORTIVE LABORATORY OR METHOD OF DIAGNOSIS
Immunodeficiencies affecting cellular and humoral immunity, n(%)	1 (2.27%)	Decreased numbers of lymphocytes and immunoglobulins, associated with opportunistic infections
Combined immunodeficiencies with associated or syndromic features	6 (13.64%)	
DiGeorge anomaly	2/5	FISH test for 22q11 deletion
Ataxia-telangiectasia	1/5	Syndromic features
Hyper-IgE syndrome	1/5	Syndromic features: NIH clinical feature scoring system
CHARGE syndrome	1/5	Syndromic features
Wiskott–Aldrich síndrome	1/5	Syndromic features
Predominantly antibody deficiency disease, n(%)	28 (63.64%)	
CVID	11/28	Low IgG and IgA and/or IgM
Specific antibody deficiency with normal Ig concentrations and normal numbers of B cells	6/28	Reduced ability to produce antibodies to specific antigens (pneumococcal polysaccharides)
X-linked agammaglobulinemia	4/28	Mutation in BTK. Severe reduction in all serum immunoglobulin isotypes with profoundly decreased or absent B cells.
Isolated IgG subclass deficiency	3/28	Reduction in one or more IgG subclass
Selective IgA deficiency	2/28	IgA decreased/absent
THI with normal numbers of B cells	2/28	IgG and IgA decreased
Diseases of immune dysregulation, n(%)	1(2.27%)	
Chediak–Higashi syndrome	1/1	Syndromic features
Congenital defects of phagocyte number and/or function	4 (9.1%)	
Congenital neutropenia	2/4	Persistent congenital neutropenia in flow cytometry. Bone marrow examination.
Chronic granulomatous disease	1/4	Dihydrorhodamine (DHR) flow cytometry test.
Cyclic neutropenia	1/4	Oscillations of neutrophils and other leukocytes and platelets.
Defects in innate immunity	2 (4.54%)	
Chronic mucocutaneous candidiasis	2/2	Phenotypic diagnosis: persistent mucocutaneous candidiasis.
Complement deficiencies, n(%)	2 (4.54%)	
C1 inhibitor deficiency	2/2	Quantitative C1 inhibitor deficiency

PID, primary immunedeficiency diseases; CVID, common variable immunodeficiency disorders; THI, transient hypogammaglobulinemia of infancy.

Until diagnosis, the median number of hospitalizations/year per patient was 0.86 (IQR = 2.28); whereas, the number of days hospitalized/year per patient was 18.8 (IQR = 49.36) and the estimated cost of hospitalizations/year per patient, without considering drugs or medical care, was $4916.5 (IQR = 15,006.6) U.S. dollars (USD). The number of visits to emergency room/year per patient prior to diagnosis was 0.92 (1.77), and the cost of visits to emergency room/year per patient was $44.78 (IQR = 123.14) USD. The number of doctor’s visits/year per patient was calculated in 15 (IQR = 11.25), and the cost of doctor’s visits/year per patient was $ 510.26 (IQR = 599.56) USD. The family expenses, monthly attributed to the disease, were estimated in $96.99 (IQR = 190.35) USD. Also, until diagnosis, patients or their relatives reported a median of 52.72 (IQR = 56.35) school/work absence days per year (Table 3).

**Table 3:** Disease burden in PID at reference hospitals in Guanajuato, Mexico.

	STUDY GROUP n = 44
Number of hospital admissions/year per patient until diagnosis, median (IQR)	0.86(2.28)
Number of days of hospitalization/year per patient until diagnosis, median (IQR)	18.8(49.36)
Cost of hospitalizations/year per patient (U.S.D)* until diagnosis, median (IQR)	$4916.5(15,006.6)
Minimum wages per day required to pay for hospitalizations in a year, median (IQR) §	1260.63(3847.83)
Visits to ER/year per patient until diagnosis, median (IQR)	.92(1.77)
Cost of visits to ER per patient per year (U.S.D)† until diagnosis, median (IQR)	$44.78(123.14)
Number of minimum wages per day to pay for visits to ER in a year, median (IQR) §	11.48(31.57)
Number of doctor’s visits/year per patient, median (IQR)	15(11.25)
Cost of doctor’s visits per patient, per year (U.S.D)‡ until diagnosis, median (IQR)	$510.26(599.56)
Number of minimum wages per day to pay for the doctor’s visits in a year §	130.83(153.73)
Family monthly expenses attributed to the disease (U.S.D), median (IQR)	96.99(190.35)
Number of minimum wages per day required to pay the family monthly expenses attributed to the disease, median (IQR) §	24.87(48.81)
School/work lost days per patient per year, median (IQR)	52.72(56.35)
Overall mortality rate since diagnosis until the time of the study, n(%)	3 (6.82%)
Death rate per year followed up, media (±SD)	0.40±0.25
Patients with complications secondary to PID, n(%)	19 (43.18%)
Number of patients treated with IVIG	20(45.45%)
IVIG number of grams per infusion per patient, median (IQR)	13.14(10.84)
Number of patients not covered by some form of health insurance	4(9.09%)

PID, primary immunodeficiency diseases; U.S.D., U.S dollars; IQR, interquartile range; ER, emergency room; IVIG, intravenous immunoglobulin.

*Costs per day of hospitalization, among institutions, range from $142 to $545.45 U.S. dollars.

† Costs per visit to emergency room range from $48.48 to 156.3 U.S. dollars.

‡ Costs of doctor’s visit per patient rage from $48.48 to $57.64 U.S. dollars.

§Minimum wage per day for general workers in Mexico is of $3.9 USD, as of January 2017.

The overall mortality rate was 6.82% (n = 3). Nineteen of the 44 patients (43.18%), were reported with complications directly or indirectly attributed to the PID (Table 3). The most commonly reported were the respiratory complications, such as bronchiectasis and chronic lung disease (n = 13/19, 68.42%), followed by chronic rhinosinusitis (n = 2/19, 10.52%). The other observed complications were: hearing impairment, anoxic encephalopathy, knee ankylosis, and short stature (n = 4/19, 21.05%).

The number of patients under intravenous immunoglobulin (IVIG) replacement therapy at the moment of the study was 20 (45.45%). The IVIG administration among the different hospitals and reference centers ranged from 3 to 4 weeks, and dosing of IVIG varied from 400 mg/dl to 1000 mg/dl. There were four patients (9.09%) that required the administration of IVIG, but were unable to receive it, because they were not covered by any kind of health insurance, neither public nor private, and were unable to afford it by themselves (Table 3).

We decided to do a comparison between number of hospital admission per year, number of days hospitalized per year, visits to emergency room per year, number of doctor’s visits per year, family monthly expenses attributed to the disease, and school/work day lost; before and after the treatment with IVIG was instigated. Among the 20 patients that were evaluated during IVIG replacement therapy (mean follow-up 3.93±2.49 years), 11 (55%) had CVID, 4 (20%) had X-linked agammaglobulinemia, 2 (10%) had Specific antibody deficiency with normal immunoglobulin concentrations, and the last 3 patients (15%) had isolated IgG subclass deficiency, chronic granulomatous disease, and Wiskott–Aldrich syndrome respectively. To further assess the disease burden of PID, we compared patients with PID before and after IVIG treatment with healthy matched controls. Table 4 shows a marked improvement and statistically significant difference in all the variables, when comparing patients with PID with themselves after the treatment with IVIG. The same was observed when comparing patients with PID before the treatment, against healthy controls. Characteristically, even when the patients with PID received IVIG, there was still an important disease burden when comparing them against healthy controls, except for number of visits to emergency room per year (Table 4).

**Table 4:** Burden of disease between patients with PID, before and after the treatment with IVIG, matched healthy controls.

	Patients with PID until diagnosis (n = 20)	Patients with PID after treatment with IVIG (n = 20)	p-value* after comparing patients with PID before and after IVIG	Healthy controls without IDP (n = 20)	p-value † after comparing healthy controls with patients with PID, before^(a)^ and after^(b)^ IVIG
Age, median (IQR)	7.58 (10.27)			8.5 (11.13)	0.289
Gender, (Male/Female)	12/8			11/9	0.75
Number of hospital admissions/year per patient, median (IQR)	0.93 (2.15)	0.09 (0.35)	0.004	0 (0)	0.000^a^
					0.046^b^
Number of days of hospitalization/year per patient, median (IQR)	20.57 (33.3)	0.22 (1.14)	0.000	0 (0)	0.000^a^
					0.043^b^
Visits to emergency room/year per patient, median (IQR)	0.17 (0.38)	0 (0.23)	0.018	0 (1)	0.026^a^
					0.9^b^
Number of doctor’s visits/year per patient, median (IQR)	18 (11.25)	3 (1.75)	0.000	1 (2.75)	0.000^a^
					0.000^b^
Family monthly expenses attributed to the disease (U.S.D), median (IQR)	93.3 (101.8)	53.35 (24.2)	0.025	10.91 (42.4)	0.000^a^
					0.000^b^
School/work lost days per patient per year, median (IQR)	57.22 (42.7)	16 (5.25)	0.000	0.5 (2)	0.000^a^
					0.000^b^

PID, primary immunodeficiency diseases; IVIG, intravenous immunoglobulin; IQR, interquartile range.

*Wilkoxon rank test.

†Mann-Whitney U test for quantitative variables, and chi-square test for nominal variables (gender).

## Discussion

As mentioned before, we included 44 patients with PID in the present study. Regarding to the gender distribution, the male predominance was similar to that previously reported from other centers [3,7,11–13]. However, in other studies, like the national surveys for PID performed in USA, in 1995, 2002, and 2007, the proportion of females with PIDs was always higher [7,11,14].

In our study, the diagnostic delay was shorter than that reported in a survey performed in 2013 at several hospitals in Mexico City [15], or the one reported in the 2007 third national survey for PID in USA [14], 2.17 vs. 12.5 vs. 12.4 years. However, in a recently reported survey at the National Institute of Pediatrics in Mexico City, and another one conducted in 2012 in Korea, the time of diagnostic delay was only of 22 and 19 months respectively [16,17]. As the diagnostic delay is still high in our study cohort and similar cohorts around the world, even with different resource settings, we should improve our efforts in order to diminish it. The creation of an educational program for health personnel and general public, could be a promising strategy to achieve this goal, as has been recently shown by a group or researchers in Aguascalientes Mexico [18].

In our study, consanguinity or inbreeding were reported in the 11.4% of the patients, almost the same proportion observed (11%) in the previously mentioned survey, at the National Institute of Pediatrics in Mexico City [16]. Characteristically, no family history of PID among patient’s relatives was reported. This finding is consistent with our previous report we made in 2013, with 26 patients [19]. Conversely, 17% of the patients in the 2007 third national survey for PID in USA and 23% of those included in the national registry of PID in Korea, had at least one family member with immunodeficiency [14,17].

In the present study, we observed that most of the patients with primary immune deficiency disease (54.55%) suffered from other chronic disease. This percentage is consistent with that reported (53%) in the 2002 second national survey for PID in USA [11]. Allergic rhinitis was the most frequent comorbidity we found in our study patients (40.91%). Regarding to other comorbidities like asthma, the percentage of patients with such disease, was very similar in our study and the second national survey for PID in USA (18.18% vs 17.8%). The high prevalence of atopic diseases among patients with PID, may suggest a link between both pathologies. Even more, allergic diseases can disguise PID symptoms, delaying diagnosis and possibly contributing to the dissemination of life threatening infections, due to the immune suppression produced by the chronic use of corticosteroids for the treatment in some patients [20].

As in other studies, antibody deficiency was the most prevalent group between PID. In our study it was reported in the 63.64% of the patients, whereas in Japan, Korea, Europe, Switzerland, and Australia, the prevalence was reported between 52.9 and 77% [17,21–24]. The group of combined immunodeficiencies was observed in the 13.64% of the patients. In third place, we found the group of congenital defects of phagocyte number and/or function. These findings, once again, are consistent with data reported in some international studies, like the European and the Australian surveys [21,24].

Until diagnosis, patients in the present study, had a median of 0.92 visits to the emergency room per year, 15 doctor’s visits/year per patient, and 0.86 hospitalizations per year related to PID. As expected, there seems to be an increase in the occurrence of such events among our patients. The high rate in the number of hospitalizations, has been reported in other studies, like the first national survey in the USA, where the 83% of the patients experienced 2 or more hospitalizations before diagnosis, and the 22% experienced 6 or more [7]. About the visits to emergency room, we could estimate a median of 0.92 visits per per patient per year to emergency services, which is more than twice the reported for the general population in USA in 2011 by the CDC, where the emergency department visits per patient per year were estimated in 0.44 [25].

An important economic impact to the health service and the individual/family with PID was shown in our study. We found that the estimated cost of hospitalizations per patient per year was $4916.5 U.S. dollars (USD), the cost of visits to emergency room per patient per year was $44.78 USD, the cost of doctor’s visits per patient per year was $ 510.26 USD, whereas the family expenses monthly attributed to the disease were estimated in $96.99 USD. At first glance, this might not seem expensive for developed economies; however, in Mexico, gross national income per capita, in 2012, was of US $10 800, compared against US$56 620 in the United States [26]. Even more, until 2014 net minimum wages per hour are very low in Mexico (<$1 USD), compared against other countries like Australia ($10 USD), United Kingdom ($9 USD), and USA ($7.3 USD) [27,28]. Related to this, as we previously mentioned in the results, the 77.27% of the study subjects in our study belong to the public health sector, which in most of the cases, are stratified into the lower social class and perceive such minimum wage. In Table 3 we tried to provide a more insightful demonstration of the economic impact of the PID in our country, calculating the minimum wages per day needed to pay for the attention the patients require. As an example, a family needs to assign 25 days of minimum wages to pay the family monthly expenses attributed to the disease of a relative with PID. The estimated school/work absence days per patient in our study was reported in 52.72 days/year, which is considerably higher to that reported in other chronic diseases like asthma. For example, in a study published in 2005, school absence days were reported in 5.81 days per year, among USA children with asthma [29]. This fact, once again, may reflect the high impact of PIDs in patients who suffer them and their relatives.

Besides the economic impact and the school/work lost days, another way to indirectly estimate the burden of a disease is measuring the complications and deaths related to it. In our study we found that 43% of patients had complications secondary to PID, which is quite similar to the proportion of patients (49%) reported in the third USA national survey. The most common complication in both studies was the chronic lung disease, 29% in our study vs. 32% at the USA survey [14]. Mortality rate in the present study was slightly slower to that reported in the national registry for PID in Korea, and the survey at National Institute in Pediatrics (6.82 vs. 9.8% vs. 13.6) [16,17]; but higher than the one reported at the German national registry for PID in 2013 (3.21%) [30].

Fortunately, it has been shown that effective treatment can reduce significantly the burden of disease, including secondary complications and death associated to PID [6]. The most common form of treatment for primary immunodeficiency diseases is intravenous immunoglobulin (IVIG) therapy. Almost 74% of patients in the third national survey of USA were currently treated with IVIG, whereas only the 45.45% of our study patients were under the same treatment. All patients with CVID and XLA at reference hospital in Guanajuato state Mexico, were under IVIG therapy. However, four patients (9%) with specific antibody deficiency with normal immunoglobulins concentrations were not receiving the IVIG replacement therapy, mainly because of the lack of some form of health insurance. Circumstances like this have been described in other reports, for example, in the first USA national survey, only 2% of people reported having any form of health insurance; nonetheless, a quarter of persons with primary immune deficiency disease report experiencing insurance problems as a result of their condition. In addition, over half report using savings, selling property or borrowing to pay for treatment [7].

To further demonstrate the impact on PID disease burden after receive proper treatment, we compared some variables before and after the patients received IVIG, like the number of hospital admissions/year per patient, the number of days hospitalized/year per patient, the number of visits to emergency room/year per patient, the number of doctor’s visits/year per patient, the family expenses monthly attributed to the disease, and the school/work absence days per year. As expected, there was an important improvement in disease burden, after the administrations of IVIG, as it has been reported in other studies [31,32]. For example, in a study performed in 2003 among Iranian patients with agammaglobulinemia, during IVIG replacement, hospitalizations due to pneumonia decreased from 0.58 to 0.05 per patient per year [33]; which is a similar behavior than the one observed in our study, where the number of hospitalizations/year diminished from 0.93 to 0.09. As we mentioned before, to further assess the disease burden of PID, we also compared patients with PID before and after IVIG treatment with healthy matched controls. We found that even when there is a considerable improvement in disease burden among patients with PID after receiving IVIG, there is still a lot to do to help our patients to get a quality of life as good as the general population. Regarding to this, the number of school/work days lost in the group of patients receiving IVIG could be decreased using subcutaneous immunoglobulin (SCIG) therapy, as has been shown in other studies evaluating health ergonomics, where de SCIG was found to be more cost effective mainly through the reduction of work or school days [34,35].

Unfortunately, there is still a lack of knowledge and insufficient research about the disease burden in PID. We are aware of the limits of the present study; however, we consider that this is a first step necessary to knowing the economic/psychosocial impact, and even the family’s perceived burden of the disease. These could be helpful, in order to establish effective interventions like convenient health programs, research, resource allocation, and the generation of better polices and practices, which in turn may alleviate, at least in part, the burden of the disease to families with PID.

## Supporting information

S1 File General database PID burden in Guanajuato, Mexico. (XLS)
